# Cost-effectiveness of portable-automated ABR for universal neonatal hearing screening in India

**DOI:** 10.3389/fpubh.2024.1364226

**Published:** 2024-08-12

**Authors:** Krushna Chandra Sahoo, Rinshu Dwivedi, Ramesh Athe, Akshay Chauhan, Shalu Jain, Rakesh Kumar Sahoo, Debdutta Bhattacharya, Kavitha Rajsekhar, Sanghamitra Pati

**Affiliations:** ^1^Health Technology Assessment in India (HTAIn), Regional Resource Hub, ICMR-Regional Medical Research Centre, Bhubaneswar, Odisha, India; ^2^Department of Health Research, Ministry of Health & Family Welfare, Health Technology Assessment in India (HTAIn), New Delhi, India

**Keywords:** digital health, hearing impairment, newborn, universal hearing screening, hearing loss

## Abstract

The World Health Organization considers Universal Neonatal Hearing Screening (UNHS) essential to global public health. Rashtriya Bal Swasthya Karyakram has included newborn hearing screening in India since 2013. The program faces human, infrastructure, and equipment shortages. First-line hearing screening with improved diagnostic accuracy is needed. The Portable Automated Auditory Brainstem Responses (P-AABR) can be used in remote areas for UNHS due to its low infrastructure needs and diagnostic accuracy. This study evaluated the cost-effectiveness of P-AABR in UNHS. We employed an analytical model based on decision trees to assess the cost-effectiveness of Otoacoustic Emission (OAE) and P-AABR. The total cost to the health system for P-AABR, regardless of true positive cases, is INR 10,535,915, while OAE costs INR 7,256,198. P-AABR detects 262 cases, whereas OAE detects 26 cases. Portable Automated ABR costs INR 97 per case detection, while OAE costs INR 67. The final ICER was 97407.69. The P-AABR device is cost-effective, safe and feasible for UNHS Rashtriya Bal Swasthya Karyakram (RBSK) programs. Beyond reducing false referrals and parent indirect costs, it detects more hearing-impaired infants. Even in shortages of skilled workers, existing staff can be trained. Thus, this study suggests integrating this device into community and primary health centers to expand UNHS coverage.

## 1 Introduction

The World Health Organization (WHO) has prioritized Universal Neonatal Hearing Screening (UNHS) as a fundamental component of global public health initiatives (1). This emphasis is rooted in the recognition that early detection and intervention for hearing impairment in newborns are crucial for the overall well-being and development of the child (2). Universal screening ensures that all newborns are tested for hearing loss shortly after birth, enabling early identification of hearing deficits, which might otherwise go unnoticed until later stages, potentially causing significant developmental delays (3). By making this screening universally accessible, WHO aims to provide every child with an equal opportunity for optimal linguistic and cognitive development, ultimately improving their quality of life (4). Additionally, early intervention can prevent further complications and associated social and economic burdens (4). This prioritization underscores the importance of addressing hearing health as an integral part of maternal and child healthcare strategies worldwide.

Infants and children born with congenital hearing impairment often face enduring challenges, including persistent speech and language difficulties, subpar academic achievements, interpersonal and emotional struggles (1–4). Timely identification, precise diagnosis, and swift intervention, however, can enable these infants with hearing loss to achieve learning outcomes on par with their peers. Hence, the 2017 World Hearing Report presents various innovative and economically efficient technological and clinical approaches with the goal of significantly enhancing the quality of life for most children facing hearing challenges (4).

A recent comprehensive review by Verma et al. demonstrated that an abnormal auditory brainstem response in neonates signified hearing impairment—prevalence ranging from 1.59 to 8.8 cases per 1,000 births (5). For children in general, the prevalence of hearing loss was found to fluctuate between 6.6 and 16.47%, as reported by the same study (5). Within this pediatric population, it was established that otitis media was the predominant cause of hearing impairment (5, 6). Moreover, it is worth highlighting that hearing impairment exhibited a higher prevalence in rural and remote areas, where the implementation of UNHS programs posed substantial challenges (7, 8).

Starting in 2013, the Rashtriya Bal Swasthya Karyakram (RBSK) in India integrated newborn hearing screening into its comprehensive childhood hearing detection program (7, 9). RBSK employs Otoacoustic Emission (OAE) for screening at healthcare facilities and utilizes Behavioral Observation Audiometry (BOA) within communities (8). At referral facilities, the confirmation of hearing loss is carried out through Brainstem Evoked Audiometry Response (BERA) (10). During mobile health team visits, a trained health worker conducts a brief questionnaire and behavioral testing as part of the RBSK screening process. Hospital-based screening for newborns follows a two-stage protocol involving Transient Evoked Oto-acoustic emissions (TEOAE) and BERA. TEOAE screening initiates at 6 weeks post-birth (11). In the event of a failed TEOAE screening, neonates are reevaluated within 3 weeks. If they continue to exhibit hearing difficulties, BERA is utilized to confirm the presence of hearing loss (12). OAE is chosen for its portability, affordability, and ease of use, but it does have a higher rate of false referrals, leading to costly BERA follow-up tests that may require sedation. Furthermore, OAE exclusively assesses the cochlear function. Challenges faced by the program include shortages in human resources, infrastructure, equipment, and a limited priority for deafness prevention. As highlighted by Sahoo et al. and Galhotra and Sahu, there is a pressing need for first-level hearing screening technology with improved diagnostic accuracy (7, 8).

The “Portable Automated ABR,” (also referred to as P-AABR), is a device based on BERA technology that screens neonates for hearing impairment with high sensitivity and specificity (13). It may be considered UNHS in settings with limited resources (14). In the P-AABR hearing screening, three electrodes are positioned on the infant’s head to gauge auditory brain waves. These electrodes are sensitive to auditory brain electrical responses elicited by stimulation. The absence of such a response indicates that the child does not possess hearing ability. Importantly, this non-invasive, battery-operated device eliminates the necessity for sedation in infants. Additionally, the device boasts a patented algorithm within its testing signal that effectively removes ambient noise, presenting another advantage compared to alternative testing systems (13, 14). Each test sent to the centralized server undergoes thorough evaluation by a trained audiologist. A favorable test result indicates that the baby has passed the screening. Conversely, if the audiologist reports a “REFER” result, the family is directed to seek assessment at an audiology center or consult with an Ear, Nose, and Throat (ENT) specialist. Notably, the advantages of the P-AABR extend to its capability to assess the hearing of newborns shortly after birth, specifically within the 0–3 day timeframe, a population that poses challenges for other hearing screening devices. This portability and minimal infrastructure requirement make the P-AABR device suitable for inclusion in UNHS initiatives in remote and underserved areas.

Among neonates categorized as “at risk,” the prevalence of congenital hearing loss in India ranges from 7 to 49.18 cases per 1,000 births (5). The cost-effectiveness of P-AABR for universal neonatal hearing screening in India is based on its ability to accurately and efficiently detect congenital hearing loss early. Early detection and intervention significantly improve language, social, and cognitive development, thereby reducing long-term costs associated with untreated hearing impairments. P-AABR’s portability and automation make it suitable for widespread use in diverse settings, enhancing accessibility and feasibility across India’s varied healthcare infrastructure (8, 14). However, it is worth noting that there exists limited empirical evidence regarding the device’s cost-effectiveness. Therefore, our study is aimed at assessing the cost-effectiveness of the P-AABR within the context of UNHS.

## 2 Methods

This study adhered to the Consolidated Health Economic Evaluation Reporting Standards (CHEERS) checklist for planning and execution, with data collected for the fiscal years 2019–20 and costs presented in Indian Rupees (INR).

### 2.1 Population, intervention, comparator, outcomes, and time horizon

The study focused on newborns aged 0–28 days population and compared the P-AABR hearing screening device (intervention) with the OAE device (comparator), primarily evaluating Quality Adjusted Life Years (QALY) and the Incremental Cost-Effective Ratio (ICER) as key outcomes over a one-year time frame.

### 2.2 Model concept, assumptions, and analysis plan

We employed an analytical model based on decision trees to assess the cost-effectiveness of neonatal hearing screening devices, specifically the OAE and P-AABR. Effectiveness in this context was defined as the ability of either device to accurately detect the hearing status of neonates with hearing loss (HL). To gauge the cost-effectiveness of these screening devices, we utilized statistical data pertaining to the annual birth rate.

The cost-effectiveness was quantified as a ratio, where the denominator represented the health benefits achieved (measured in years of life and prevention of premature births), and the numerator encompassed the associated costs, factored against quality-adjusted life years (QALY). Finally, we computed incremental cost-effectiveness ratios (ICER). Newborns who tested positive, regardless of whether it was a true or false result when compared to the BERA, were subjected to diagnosis. Conversely, newborns who tested negative were discharged and not subjected to further follow-up (terminal node) (Supplementary File 1).

Every device comprises four branches and end nodes. The cost associated with screening and confirming the diagnosis of newborns with true positive hearing loss (HL) (Branch A/A’). The cost of screening for newborns with false negative HL (Branch B/B′). The cost of screening and confirming the diagnosis of newborns with false positive results indicating normal hearing (Branch C/C′). The cost of screening newborns with true negative results indicating normal hearing (Branch D/D′).

Each device’s total cost was equal to the sum of these four branches. We calculated the expected efficacy of each device by multiplying the number of newborns entering the model by the prevalence and sensitivity of each device. This model’s primary inputs were the prevalence of HL in India, the sensitivity and specificity of screening devices, the cost of screening, and the definitive diagnosis of each newborn. We evaluated the implementation cost of P-AABR and OAE devices for newborn hearing screening using a decision tree model with a 1-year time horizon. The perspective was societal and health system-based.

### 2.3 Study settings and participants

Six facilities, including District Early Intervention Centers (DEIC) under RBSK in Odisha state (three coastal and three non-coastal), where OAE devices were implemented, were randomly selected to collect cost data (8). The prevalence of congenital hearing loss among infants was based on actual epidemiological statistics from India (5). Using a pre-designed questionnaire, all information regarding OAE-related health system expenditures, such as cost and OAE detection rates, was gathered. The cost information for the P-AABR was obtained from the manufacturer, while P-AABR detection rates were sourced from a previous feasibility study (14), and diagnostic validity information was obtained from the primary study.

### 2.4 Measurement and valuation of resources and costs

We compiled the expenses within the healthcare system related to the deployment of P-AABR, OAE and BERA. This encompassed various elements, including human resources, medical equipment and supplies, non-consumable items, maintenance, and utility costs.

The human resource costs covered the salaries of all screening personnel, including both medical and non-medical staff. The inventory for medical supplies and consumables encompassed screening equipment, with particular attention to the expenses linked to OAE and P-AABR devices. Consumables encompassed both medical items, such as drugs and reagents, and non-medical items like stationery, with comprehensive data on their quantity and pricing. Furniture and technical equipment, such as computers installed in screening centers, constituted non-consumables. The maintenance budget was expressed as a proportion of the total annual budget. Also documented was the allocation of utility resources such as electricity and water to the program.

We collected data from the patient’s perspective regarding out-of-pocket expenditure (OOPE). Since most of the expenses are covered by the Janani Shishu Suraksha Karyakaram (JSSK), RBSK programs, and various government-funded schemes related to maternal and child healthcare, patients generally do not have to bear any direct OOPE. However, in cases of emergencies, patients might still incur some travel expenses and potential wage loss due to their absence from work or reduced working hours until they receive proper referrals. To account for this, we considered wage loss as an indirect indicator of OOPE.

To assess infants’ quality of life (QoL), we used a descriptive system that used the Infant Health-related Quality of Life Instrument (IQI) via a mobile app. The IQI assessed seven key health attributes: sleeping, feeding, breathing, stooling/pooping, mood, skin condition, and interaction (15). To compute these average scores, we followed the methodology outlined by Abram et al., who adapted values from the Health Utilities Index Mark 3 (16). This index assigns various health states to the attributes of hearing and hearing aid usage. In our study, we initially screened one hundred thousand neonates using both P-AABR and OAE tests. These instruments classified neonates as either “pass,” indicating normal hearing (NHL), or “refer,” indicating abnormalities or hearing loss (HL). We defined HL as permanent congenital bilateral hearing loss greater than 35 dB, assuming the screening was performed by an audiologist. Supplementary File 2 contains the detailed hearing impairment screening pathway.

## 3 Results

Table 1 presents the clinical parameters alongside the average Quality of Life (QoL) scores, which were derived from our primary dataset. Table 2 provides a breakdown of the expenses associated with conducting screenings for P-AABR, OAE, and BERA. Completing an infant’s hearing test with P-AABR typically requires about 15 min, while OAE takes around 10 min, and BERA consumes approximately 90 min. With a 260-day work year, it is possible to test infants annually with OAE (*n* = 9,360), P-AABR (*n* = 6,240), and BERA (*n* = 1,040).

**Table 1:** Clinical parameters and quality of life (QoL).

Clinical parameters	Value
Prevalence of HL per 1,000	5
Sensitivity of P-AABR	100%
Specificity of P-AABR	97%
Positive predicted values (PPV) for P-AABR	52%
Negative predictive values (NPV) for P-AABR	100%
Sensitivity of OAE	69%
Specificity of OAE	68%
Positive predicted values (PPV) for OAE	7%
Negative predictive values (NPV) for OAE	98%
QoL weights	
Normal hearing (may have other health problems)	0.95
HL (unilateral and bilateral)	0.77
Unilateral HL	0.85
Bilateral HL	0.69
Cohort and case detection	
Neonatal population (Cohort)	100,000
Cases detected by portable automated ABR	262
Cases detected by OAE	38
Life expectancy (2012–2016) at birth	69.2

**Table 2:** Screening cost for implementation of P-AABR, OAE, and BERA.

Screening	P-AABR	OAE	BERA
The device’s lifespan in years	6	6	6
The average duration of test for one new-born in minutes	0.25	0.17	1.5
Mean screening of new-born in 1 day	24	36	4
Average number of working days in a year	260	260	260
Mean screening of new-borns (No. of cases per year)	6,240	9,360	1,040
Cost (INR)			
Human resource	421,000	421,000	1,291,000
Medical consumables	104,520	140,400	174,720
Non-medical consumables	0	0	4,000
Medical equipment	74,736	58,883	187,107
Non-medical equipment (including building/space)	0	0	139,354
Overheads	2,400	2,400	30,000
Total Cost (INR)	602,656	622,656	1,826,181
Unit cost for hearing screening (INR)	97	67	1756

The financial aspects related to the implementation of OAE, P-AABR, and BERA indicate that OAE costs INR 421,000, P-AABR also amounts to INR 421,000, while BERA incurs an approximate cost of INR 129,000. A comprehensive breakdown of these expenses is given in Supplementary File 3. Furthermore, the medical consumables cost for P-AABR is INR 104,520, for OAE it is INR 140,400, and for BERA, it stands at INR 174,720. Supplementary File 4 details the total cost of implementing P-AABR and OAE, including both medical non-consumables and consumables. Supplementary File 5 details the annual costs for implementing OAE, P-AABR, and BERA, including both non-consumables and consumables. The cost of human resources for the treatment and rehabilitation of hearing impairment was INR 630,000. Additionally, the procedural cost per child for treatment and rehabilitation was INR 77,774. The combined total cost for both human resources and procedural expenses for the treatment and rehabilitation of hearing impairment is detailed in Supplementary File 6 and sums up to INR 707,774. The mean transportation expenses per hearing screening visit differ across various healthcare facility levels. Specifically, at medical college hospitals, it averages 440 INR, at district or sub-divisional hospitals it stands at 300 INR, at community health centers it amounts to 207 INR, and at primary health centers, it is as low as 99 INR.

**Figure 1:**
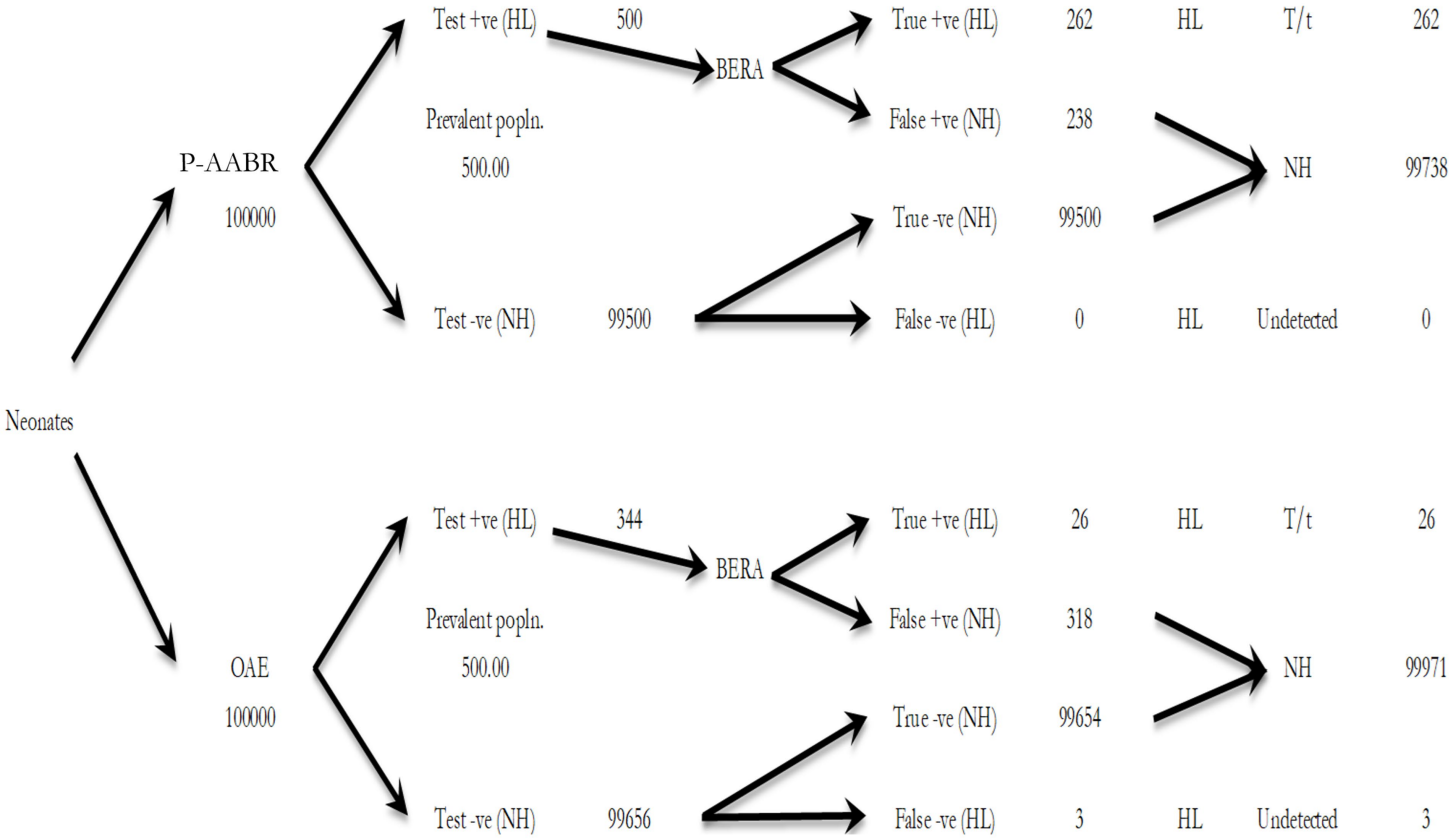
The decision tree model for the prevalent target population.

Table 3 provides an estimation of the annual health system costs in INR for human resources and consumables necessary to implement OAE, P-AABR, and BERA. According to the decision tree analysis, if hearing screening were conducted on 100,000 infants with a prevalent cohort population of 500 utilizing the P-AABR device, the following outcomes are anticipated: 500 newborns would be accurately identified as having positive hearing loss (HL), while 99,738 newborns would be correctly identified as having negative HL or normal hearing (NH). Among the 500 cases referred to the gold standard BERA, 262 newborns would be correctly identified as having HL, 238 would be incorrectly identified as not having HL, and there would be no cases of HL under-detection. Similarly, employing the OAE device is expected to yield the following outcomes: 344 newborns will be accurately identified as having positive hearing loss (HL), and 99,971 newborns will be correctly identified as having negative HL or normal hearing (NH). Out of the 344 cases referred to the gold standard BERA, 26 newborns will be correctly identified as having HL, 318 will be incorrectly identified as not having HL, and there will be 3 cases of under-detected HL. The decision tree model for the prevalent target population is provided in Figure 1.

**Table 3:** Estimation of annual health system cost (INR) for human resources and consumables for implementation of OAE, P-AABR, and BERA.

Human resources	Monthly Salary	Time spends exclusively for screening (in hour per day)	Overall working hours (monthly)	Overall working hours (yearly)	Monthly time on screening (in hours)	Apportioning statistic	Monthly cost to system	Annual cost (in INR) OAE	Annual cost (in INR) portable automated ABR	Annual cost (in INR) BERA
Audiologist	50,000	8	176	2080	176	1	50,000	0	0	600,000
Staff nurse	15,000	8	176	2080	176	1	15,000	180,000	180,000	180,000
Data entry operator/technician	18,000	8	176	2080	176	1	18,000	216,000	216,000	216,000
Pediatrician/Anesthesiologist	90,000	2	176	2080	44	0.25	22,500	0	0	270,000
Post service training per person								25,000	25,000	25,000
Medical consumables (electrodes and sedatives)								140,400	104,520	174,720
Non-medical (stationery and cartridge)								0	0	4,000
Soundproof room and building								0	0	412,800
Electricity and water per annum								2,400	2,400	30,000
Direct cost total (health system)								563,800	527,920	1,955,720
Cross-tally								563,800	527,920	1,955,720

Non-consumables	OAE	PA-ABR	BERA	Expected life	Units	Unit price	Total cost	Discount factor	Annual maintenance rate	Annualization factor	EUAC capital	Annual maintenance cost	Present worth maintenance	Total annual cost
Medical								0.03	0.05	0.1846	60917.18	16,500	13818.49	74,736
	Portable automated ABR													
								0.03	0.05	0.1846	47995.35	13,000	10887.30	58,883
	**OAE**													
Device cost	260,000	330,000	826,184	6	1			0.03	0.05	0.1846	152511.50	41309.2	34595.80	187,107
**Non-medical**														**BERA**
Computer	0	0	152,000	5	1	20,000	20,000	0.03	0.05	0.2184	4367.09	1,000	862.61	5229.70
Air conditioner	0	0		10	2	36,000	72,000	0.03	0.05	0.1172	8440.60	3,600	2678.74	11119.33
Printer	0	0		5	3	3,000	9,000	0.03	0.05	0.2184	1965.19	450	388.17	2353.37
Table	0	0		5	2	9,000	18,000	0.03	0.05	0.2184	3930.38	900	776.35	4706.73
chair	0	0		5	3	3,000	9,000	0.03	0.05	0.2184	1965.19	450	388.17	2353.37
stool	0	0		5	2	1,000	2000	0.03	0.05	0.2184	436.71	100	86.26	522.97
Bed	0	0		5	1	8,000	8,000	0.03	0.05	0.2184	1746.84	400	345.04	2091.88
Almirah	0	0		7	1	10,000	10,000	0.03	0.05	0.1605	1605.06	500	406.55	2011.61
Tube lights	0	0		2	2	2000	4,000	0.03	0.05	0.5226	2090.44	200	188.52	2278.96
	0	0											**Total**	32667.92
Soundproof room	0	0	408,000	5	1	408,000	408,000	0.03	0.05	0.2184	89088.67	20,400	17597.22	106685.88
	0	0											**BERA**	139353.80

Table 4 summarizes the cost-effectiveness analysis results for OAE and P-AABR devices, including the number of cases detected, the cost per detected case, and the total cost. Children without hearing loss have a QALY of 0.95, while children with hearing loss (HL) have a QALY of 0.77.

**Table 4:** Final outcomes for cost-effective analysis with OAE and P-AABR devices for number of cases detected, per case detection and total cost.

Health systems (portable automated ABR/OAE + BERA)				
Device	Costs (with cochlear implant)	QALYs (with cochlear implant)	Costs (with hearing aid)	QALYs (with hearing aid)
Portable automated ABR	332678772.18	6574000.00	193869248.38	6574000.00
OAE	39111934.20	6573966.33	25385478.72	6573966.33
Difference	293566837.98	33.67	168483769.65	33.67
ICER		8718388.30		5003654.15
Societal (portable automated ABR/OAE + BERA)				
Portable automated ABR	413235150.18	6574000.00	274425626.38	6574000.00
OAE	119543069.08	6573966.33	105816613.60	6573966.33
Difference	293692081.11	33.67	168609012.78	33.67
ICER		8722107.79		5007373.64
	Cost of detecting 1 case	Total cost (HS) w/o T/t	Cases detected	Cost per case detection
	With portable automated ABR	10,535,915	262	40,228
	With OAE	7,256,198	26	280,173
Final ICER	97407.69			

The total cost to the health system for P-AABR, regardless of true positive cases, is INR 10,535,915, while OAE costs INR 7,256,198. P-AABR detects 262 cases, whereas OAE detects 26 cases. Portable Automated ABR costs INR 97 per case detection, while OAE costs INR 67. The final ICER was 97407.69 (Table 4).

## 4 Discussion

The effectiveness of P-AABR compared to OAE for neonatal hearing screening in India reveals significant advantages. P-AABR directly measures the neural pathways of hearing, offering higher accuracy in detecting hearing impairments, including auditory neuropathy spectrum disorder, which OAE may miss (13, 14). While OAE is quicker and less expensive, its sensitivity to outer and middle ear conditions can lead to higher false-positive rates. In contrast, P-AABR’s comprehensive assessment reduces the likelihood of missed diagnoses, making it a more reliable method for neonatal hearing screening in diverse and often resource-limited settings across India (13, 14). This effectiveness supports the integration of P-AABR into standard screening protocols to enhance early detection and intervention outcomes.

The P-AABR device costs more per newborn to screen than the OAE device. UNHS benefits from P-AABR’s large increase in accurately detected cases, which lowers case costs and healthcare system and society costs. P-AABR’s diagnostic accuracy and cost-effectiveness make it the best choice over OAE for UNHS. The P-AABR method had fewer false positives, meaning fewer healthy newborns were misdiagnosed with hearing problems. This lowers direct, indirect, and intangible costs and reduces stress and anxiety for newborn families. P-AABR is a viable alternative to OAE, reducing referred cases and newborn screening costs. Despite its higher upfront costs, P-AABR screening is more efficient and cost-effective than OAE.

Early identification of hearing issues in children is the cornerstone of effective intervention and support (17, 18). It allows for the timely implementation of measures such as hearing aids, cochlear implants, or educational accommodations, which are crucial for optimizing language and speech development during the critical early years of a child’s life (19, 20). Without early screening and detection, hearing problems may go unnoticed, potentially leading to delayed diagnosis and missed opportunities for intervention (14, 21). This delay can affect a child’s education, social and emotional well-being, and life quality (18). Additionally, it can strain families and healthcare systems. Therefore, the proactive approach of screening and early detection not only benefits the child but also contributes to more inclusive and supportive communities, ensuring that children with hearing issues have every opportunity to thrive and reach their full potential.

In low-and middle-income countries (LMICs), UNHS faces several key barriers that pose significant challenges to its successful implementation (22–26). Firstly, limited access to healthcare services and infrastructure in many LMICs hinders the establishment of comprehensive UNHS programs. Inadequate facilities and a shortage of trained healthcare personnel can lead to delays in screening or the absence of screening altogether (8, 27). Secondly, financial constraints are a major barrier, as many families in LMICs may struggle to afford the cost of screening and follow-up services, especially in the absence of universal healthcare coverage (28, 29). Thirdly, there is a notable lack of awareness and education regarding the importance of early hearing detection and intervention among both healthcare providers and parents. Cultural beliefs and stigma surrounding hearing loss can also influence decisions related to screening and intervention (8, 30). To overcome these barriers and ensure equitable access to UNHS, concerted efforts are required, including increased investment in healthcare infrastructure, public awareness campaigns, training for healthcare workers, and the development of cost-effective screening intervention strategies in the context of LMICs.

There were significant challenges, both in terms of demand and supply, associated with the current hearing screening technologies in LMICs (8, 31–33). These challenges underscored the necessity for a portable and user-friendly technology capable of achieving improved diagnostic accuracy during the initial screening stage at primary healthcare facilities for detecting hearing impairment (8, 34). The P-AABR is regarded as a portable, user-friendly, clinically efficient, and cost-effective device. It minimizes the need for extensive manpower, as it can be operated by junior staff nurses rather than relying heavily on audiologists (14). Given the substantially higher societal costs associated with untreated deaf infants, the most sustainable long-term approach may involve identifying the total number of missed cases, specifically those left untreated, through implementation at primary healthcare facilities.

The current hearing screening program under RBSK in India is primarily offered by District Early Intervention Centers (DIECs) at tertiary healthcare facilities. However, given the prevalence of non-institutional deliveries in the country, a significant number of infants do not undergo early hearing screening (7, 8, 35). This includes cases in community health centers, primary health centers, and instances of community deliveries, all of which remain unaddressed in terms of hearing screening. Moreover, many tertiary care facilities and DIECs are situated at considerable distances from these communities, resulting in substantial travel expenses for parents bringing their infants for screening. This travel cost is further compounded by potential wage loss, exacerbating the financial burden on families. To make hearing screening services more accessible and cost-effective for all, we propose the implementation of hearing screening services at community health centers and primary health centers. This approach would not only reduce indirect costs but also address intangible expenses associated with travel and lost wages. Additionally, this study suggests exploring the possibility of extending screening services to outreach areas as an alternative to the existing approach under RBSK. Furthermore, to enhance the UNHS, the P-AABR device could be integrated into all delivery points.

When considering a societal perspective, it is important to recognize that the lifetime costs associated with both treated and untreated hearing loss can vary significantly. This is particularly evident in LMICs, where healthcare resources are limited for both treated and untreated cases (8, 27). Untreated infants incur higher lifetime costs due to later expenses. Undiagnosed cases go untreated throughout a person’s life, resulting in low healthcare costs (22, 23). Missing a case has a high economic and health-related quality of life cost, emphasizing the importance of early intervention. Thus, the strategy should prioritize reducing undetected cases. Our field study found challenges recruiting audiologists, especially in remote areas. The P-AABR device can solve the skilled labor shortage because other staff members can operate it with basic training and supervision. Compared to OAE, the device’s high sensitivity and specificity reduce false referrals and improve child hearing loss detection.

The cost-effectiveness of P-AABR for universal neonatal hearing screening in India has important policy and practical implications. By incorporating P-AABR into national health policies, India can more effectively address congenital hearing loss, resulting in significant improvements in language, social, and cognitive development for affected infants. This, in turn, may reduce the long-term economic burden on the public health system caused by untreated hearing impairments. The technology’s portability and automation make it suitable for widespread use, even in remote and resource-constrained settings, promoting equitable healthcare access. However, challenges such as providing adequate training for healthcare workers, maintaining device quality, and securing ongoing funding for the program must be addressed. To ensure scalability and sustainability, mitigation strategies include collaboration between the public and private sectors, ongoing professional development programs, and a phased implementation approach.

Additionally, in analyzing the regions of India where newborn hearing screening is more or less prevalent, it is crucial to highlight the disparities in screening coverage across different states and territories. This regional analysis will provide a comprehensive understanding of the current landscape, identifying areas with successful implementation and those lagging behind. Such insights are valuable for policymakers, enabling them to allocate resources more effectively and devise targeted interventions to ensure uniform access to newborn hearing screening across the country. Understanding these regional variations can help bridge the gap and promote early detection and intervention for hearing impairments in newborns nationwide.

In conclusion, the P-AABR device boasts portability, safety, simplicity in technology, and remarkable diagnostic accuracy, making it a cost-effective option that can seamlessly integrate into existing RBSK programs for UNHS. Its benefits extend beyond reducing false referrals and alleviating the indirect costs borne by parents; it also excels in detecting a larger number of infants with hearing loss. Even in situations where there is a shortage of skilled professionals, it can be readily taught to existing personnel. Thus, this study recommends the integration of this device into community health centers and primary health centers to expand UNHS coverage.

## Data availability statement

The datasets presented in this study can be found in online repositories. The names of the repository/repositories and accession number(s) can be found in the article/Supplementary material.

## Ethics statement

The studies involving humans were approved by the Institutional Ethical Committee of the ICMR-Regional Medical Research Centre, Bhubaneswar, Odisha, India. The studies were conducted in accordance with the local legislation and institutional requirements. Written informed consent for participation in this study was provided by the participants’ legal guardians/next of kin. Written informed consent was obtained from the individual(s), and minor(s)’ legal guardian/next of kin, for the publication of any potentially identifiable images or data included in this article.

## Author contributions

KS: Conceptualization, Data curation, Formal analysis, Investigation, Methodology, Project administration, Resources, Software, Supervision, Validation, Visualization, Writing – review & editing. RD: Conceptualization, Data curation, Formal analysis, Investigation, Methodology, Project administration, Software, Supervision, Validation, Writing – review & editing. RA: Conceptualization, Data curation, Formal analysis, Investigation, Methodology, Software, Supervision, Writing – review & editing. AC: Data curation, Formal analysis, Methodology, Software, Validation, Writing – review & editing. SJ: Conceptualization, Funding acquisition, Methodology, Visualization, Writing – review & editing. RS: Data curation, Investigation, Project administration, Writing – review & editing. DB: Conceptualization, Data curation, Funding acquisition, Investigation, Project administration, Resources, Visualization, Writing – review & editing. KR: Conceptualization, Funding acquisition, Project administration, Resources, Visualization, Writing – review & editing. SP: Conceptualization, Data curation, Funding acquisition, Investigation, Methodology, Project administration, Resources, Supervision, Visualization, Writing – review & editing.
