# Supplementary material for: Cost-effectiveness of portable-automated ABR for universal neonatal hearing screening in India
Source: Front Public Health. 2024 Aug 12;12:1364226. doi: 10.3389/fpubh.2024.1364226 (PMC11345169; doi:10.3389/fpubh.2024.1364226)
Supplement: Supplementary file 1 [file Data_Sheet_1.docx]

Supplementary Material

# Supplementary figures

Supplementary file 1. The decision-tree model for the hearing screening

**
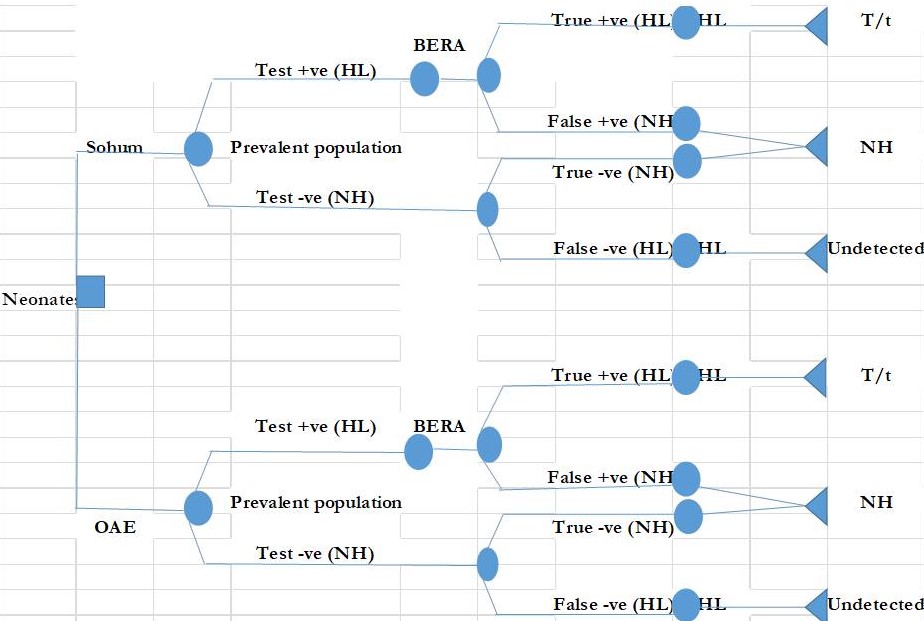
**

Supplementary file 2. Pathway for the screening of hearing impairment


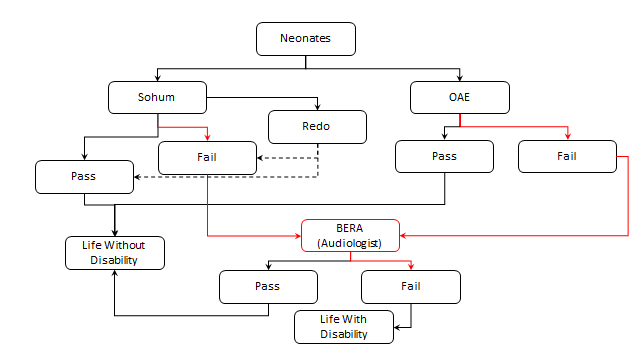


## Supplementary Tables

# Supplementary file 3. Human resources cost for implementation of OAE, P-AABR and BERA

| **Human Resources for screening and diagnosis** | **Monthly Salary** | **Number of working days per month** | **Time spent exclusively for screening (in hour per day)** | **Time on screening in hours (Monthly)** | **overall working hours (Yearly)** | **Apportioning statistic** | **Cost to system (Monthly)** | **Annual cost OAE** | **Annual cost ‘Portable Automated ABR’** | **Annual cost BERA** |
| --- | --- | --- | --- | --- | --- | --- | --- | --- | --- | --- |
| Staff Nurse | 15000 | 22 | 8 | 176 | 2080 | 1 | 15000 | 180000 | 180000 | 180000 |
| Technician | 18000 | 22 | 8 | 176 | 2080 | 1 | 18000 | 216000 | 216000 | 216000 |
| Audiologist | 50000 | 22 | 8 | 176 | 2080 | 1 | 50000 | 0 | 0 | 600000 |
| Paediatrician or Anaesthesiologist | 90000 | 22 | 2 | 44 | 2080 | 0.25 | 22500 | 0 | 0 | 270000 |
| Post service training per person (staff nurse, technician, and audiologist) |  |  |  |  |  |  |  | 25000 | 25000 | 25000 |
| Total (screening and diagnosis cost) |  |  |  |  |  |  |  | 421000 | 421000 | 1291000 |

Supplementary file 4. Total medical non-consumables and consumables cost for implementation of P-AABR and OAE

| **Items** | **Cost (INR)** | **Description** | **Per unit** | **Per baby** | **Per 1000 babies** |
| --- | --- | --- | --- | --- | --- |
| **Portable Automated ABR** |  |  |  |  |  |
| Device cost | 330000 | per unit |  | NA | NA |
| Annual maintenance cost for device | 9000 | per device |  | NA | NA |
| one ear tip | 5 | used for 20 after cleaning | 0.25 | 0.25 | 250 |
| Total cost for testing with non-disposable electrodes |  |  |  | 9 | 9017 |
| Disposable electrode | 5 | 3 for one baby | 15 | 15 | 15000 |
| Disposable electrode lead | 3600 | set of 3 | 1 | 1 | 1000 |
| one ear tip | 5 | used for 20 after cleaning | 0.25 | 0.25 | 250 |
| Earphones | 1 | 15000 | 0.5 | 0.5 | 500 |
| Total cost for testing with disposable electrodes |  |  |  | 16.75 | 16750 |
| **OAE** |  |  |  |  |  |
| OAE Device cost | 260000 |  |  |  |  |
| Annual maintenance cost for device | 26000 |  |  |  |  |
| OAE Probe | 72000 | life would be 1-1.5 |  |  |  |
| Disposable ear trips | 15 |  | 15 | 15 | 15000 |
| Total cost of testing with disposable probe |  |  | 15 | 15 | 15000 |

Supplementary file 5. Annual non-consumables and consumables cost for implementation of OAE, P-AABR and BERA

| **Heads** | **Expected Life** | **Units** | **Unit Price** | **Total cost** | **Discount factor (DF)** | **Annual maintenance rate (AMR)** | **Annualization Factor (F)** | **EUAC Capital** | **Annual Maintenance cost (AMC)** | **Present worth maintenance** | **Total annual cost** |
| --- | --- | --- | --- | --- | --- | --- | --- | --- | --- | --- | --- |
| **OAE** | | | | | | | | | | | |
| Medical (device cost) [A] | 6 | 1 | 320000 |  | 0.03 | 0.05 | 0.1846 | 59071 | 16000 | 13400 | 72471 |
| Medical Consumables (Disposable ear trips and others) [B] | One time | 1 | 15 | single use for per baby | | | | | | | 140400 |
| Total [A+B] |  |  |  |  |  |  |  |  |  |  | **212871** |
| **Portable Automated ABR** | | | | | | | | | | | |
| Medical (device cost) [A] | 6 | 1 | 330000 |  | 0.03 | 0.05 | 0.1846 | 60917 | 16500 | 13818 | 74736 |
| Medical (disposable electrode, lead, ear trips [B] | One time | 1 | 16.75 | single use for per baby | | | | | | | 104520 |
| Total [A+B] |  |  |  |  |  |  |  |  |  |  | **179256** |
| **BERA** | | | | | | | | | | | |
| Medical (device cost) [A] | 6 | 1 | 826184 |  | 0.03 | 0.05 | 0.1846 | 152511 | 41309.2 | 34596 | 187107 |
| Non-medical [B] | | | | | | | | | | | |
| *Computer* | 5 | 1 | 20000 | 20000 | 0.03 | 0.05 | 0.218 | 4367 | 1000 | 863 | 5230 |
| *Air conditioner* | 10 | 2 | 36000 | 72000 | 0.03 | 0.05 | 0.117 | 8441 | 3600 | 2679 | 11119 |
| *Printer* | 5 | 3 | 3000 | 9000 | 0.03 | 0.05 | 0.218 | 1965 | 450 | 388 | 2353 |
| *Table* | 5 | 2 | 9000 | 18000 | 0.03 | 0.05 | 0.218 | 3930 | 900 | 776 | 4707 |
| *Chair* | 5 | 3 | 3000 | 9000 | 0.03 | 0.05 | 0.218 | 1965 | 450 | 388 | 2353 |
| *Stool* | 5 | 2 | 1000 | 2000 | 0.03 | 0.05 | 0.218 | 437 | 100 | 86 | 523 |
| *Bed* | 5 | 1 | 8000 | 8000 | 0.03 | 0.05 | 0.218 | 1747 | 400 | 345 | 2092 |
| *Almirah* | 7 | 1 | 10000 | 10000 | 0.03 | 0.05 | 0.161 | 1605 | 500 | 407 | 2012 |
| *Tube lights and others* | 2 | 2 | 2000 | 4000 | 0.03 | 0.05 | 0.523 | 2090 | 200 | 189 | 2279 |
| *Soundproof room* | 5 | 1 | 408000 | 408000 | 0.03 | 0.05 | 0.2184 | 89089 | 20400 | 17597 | 106686 |
| Total [B] |  |  |  |  |  |  |  |  |  |  | 139354 |
| Consumables | | | | | | | | | | | |
| *Testing and general supplies (cotton, gel, conductive and sanitizers)* |  |  |  |  |  |  |  |  |  |  | 11440 |
| *If disposable electrodes use* |  |  |  |  |  |  |  |  |  |  | 18720 |
| *Sedatives (Triclofos)* |  |  |  |  |  |  |  |  |  |  | 156000 |
| Total [C] |  |  |  |  |  |  |  |  |  |  | 186160 |
| Non-medical (ink and paper and other stationary items) [D] |  |  |  |  |  |  |  |  |  |  | 4000 |
| Total [A+B+C+D] |  |  |  |  |  |  |  |  |  |  | **516621** |

Supplementary file 6. Human resources and procedural cost for treatment and rehabilitation of hearing impairment

| **Human resource** | **Monthly Salary** | | | **Number of working days (Monthly)** | **Time spends exclusively for screening (in hour per day)** | **Time on screening in hours (Monthly)** | **Overall working hours (in a year)** | **Apportioning statistic** | **Monthly cost to system** | **Treatment cost (for only positive cases)** | |
| --- | --- | --- | --- | --- | --- | --- | --- | --- | --- | --- | --- |
| ENT Specialist | 90000 | | | 22 | 2 | 44 | 2080 | 0.25 | 22500 | 270000 | |
| Counsellor | 18000 | | | 22 | 8 | 176 | 2080 | 1 | 18000 | 216000 | |
| Therapist | 32000 | | | 22 | 3 | 66 | 2080 | 0.375 | 12000 | 144000 | |
| Total [A] |  | | |  |  |  |  |  |  | 630000 | |
| **Procedural cost (per child)** | **Cost** | **Expected Life** | **Units** | **Discount factor (DF)** | **Annual maintenance rate (AMR)** | **Annualization Factor (F)** | **EUAC Capital** | **Annual Maintenance cost (AMC)** | **Present worth maintenance** | | **Total cost (Yearly)** |
| Hearing Aid | 20000 | 5 | 1 | 0.03 | 0.05 | 0.22 | 4367 | 1000 | 863 | | 5230 |
| Cochlea Implant | 550000 | Life time | 1 | 0.03 | 0.05 | 0.03 | 18967 | 27500 | 3577 | | 22545 |
| Therapy cost (Lump sum) | 50000 | 1 |  | 0 | 0 | 0 | 0 | 0 | 0 | | 50000 |
| Total (PC) [B] |  |  |  |  |  |  |  |  |  | | 77774 |
| Grand Total [A+B] |  |  |  |  |  |  |  |  |  | | 707774 |
